# Improving Osteoporosis Screening in Parkinson's Disease and Related Conditions—Think FRAGILE

**DOI:** 10.1111/ajag.70234

**Published:** 2026-09-10

**Authors:** Christopher Coppin, Ayush Jha, Simon J. G. Lewis

**Affiliations:** ^1^ Parkinson's Disease Research Clinic, Macquarie Medical School, Macquarie University Sydney Australia; ^2^ Imperial College School of Medicine, Imperial College London London UK

## Abstract

**Objectives:**

Patients living with Parkinson's disease (PD) and related disorders are at increased risk of osteoporosis and fractures. However, bone health remains under‐assessed in clinical practice. The aim of this study was to evaluate whether the FRAGILE tool that we previously developed improved osteoporosis screening and referral practices in a PD and related diseases clinic.

**Methods:**

We performed an audit of 283 outpatient consultation letters evaluating the use of the FRAGILE tool for screening osteoporosis risk.

**Results:**

Following implementation of the FRAGILE tool our referral rate for osteoporosis screening improved from 23% to 48% of at‐risk patients. Specifically, marked improvements were seen in referrals for patients with osteopenia, falls and fractures, but factors such as osteoporosis‐associated medications and medical comorbidities were still often overlooked.

**Conclusions:**

Osteoporosis screening referrals improved after introduction of the FRAGILE tool in clinical practice. Further efforts are required to address less overt risk factors.

## Impact Statements

Patients living with Parkinson's disease and related conditions are at increased risk of osteoporosis and its related morbidity. This study demonstrates that the use of a simple mnemonic memory tool, in this case FRAGILE, is able to dramatically improve the detection of and response to osteoporosis risk factors.

## 1 Introduction

It is well established that patients with Parkinson's disease (PD) are at over twice the risk of developing osteoporosis and sustaining fractures compared with age‐matched controls [1]. The risk of falls may also be increased in patients with other related syndromes (dementia with Lewy bodies—DLB, progressive supranuclear palsy—PSP, prodromal Lewy body disease, isolated REM Sleep Behaviour Disorder—iRBD and multiple system atrophy—MSA) [2], prompting the need for regular screening and treatment in this population.

Previously, our team has reported a significant gap in osteoporosis risk assessment and referral within a Parkinson's and related diseases clinic in Australia [3]. In our initial audit, only 23% of at‐risk patients were directed to their general practitioner (GP) for bone health screening. Given the substantial morbidity arising from fractures in this population, we developed a pragmatic cognitive prompt composed of seven factors that are important to osteoporosis risk in clinical practice. We arranged these seven core factors into a mnemonic, FRAGILE:

- *F*ractures—history of prior minimal trauma fractures or known osteopenia.
- *R*isky medications—for example, taking glucocorticoids ≥ 4 months, androgen deprivation therapy, aromatase inhibitors
- *A*ctivity—encourage exercise as a preventative intervention in all patients
- *G*ait—history of falls or imbalance
- *I*nstitutionalisation—documented frailty or living in an institution (e.g., nursing home)
- *L*ong‐term comorbidities—for example, diabetes, rheumatoid arthritis and inflammatory bowel disease
- *E*ducation and documentation—ensure osteoporosis advice is given and recorded

This FRAGILE mnemonic tool was developed with reference to the Royal Australian College of General Practitioners (RACGP) guidelines [4] to ensure relevance to the local clinical population. In contrast to other tools such as FRAX and BONE PARK [5], FRAGILE does not require a weight or height from the patient, which improves applicability to a busy sub‐speciality clinic. It is intended as a memory aid for risk factors, rather than a strict risk calculator like FRAX. Clinicians were able to use the tool how they saw fit, whether including it in a letter template or referring to it only as required. To assess whether having the FRAGILE tool available in our clinic was associated with more effective screening in clinical practice, we repeated our audit after 1 year using the same methodology.

## 2 Methods

We screened 283 sequential consultation letters from a 3‐month period in our subspecialty clinic, which included patients living with PD (*n* = 233), DLB (*n* = 25), PSP (*n* = 11), iRBD (*n* = 9) and MSA (*n* = 2), noting that three patients were reviewed twice over that period. Most consultations were follow‐up patients. The letters were assessed by one author to identify whether any of the risk factors were present from the FRAGILE tool described above. Where a risk factor was present, it was also determined whether appropriate action was taken, which in the context of our practice most commonly meant referring back to their GP for further investigation for osteoporosis or for the initiation of treatment, which we had decided on as the best course of care in our initial audit [3].

The referral information and risk factors were recorded only if explicitly documented in the clinic letter. As such, this audit is focused on measuring whether appropriate steps were taken after a risk factor was identified, as this is the area we felt needed improvement after our initial 2024 baseline audit, which occurred 12 months prior and similarly reviewed 3 months of sequential patient letters [3].

## 3 Results

Of the 283 patients screened, 132 were identified as having modifiable risk factors for osteoporosis. Of these, 51 patients were already being managed appropriately. Of the remaining 81 patients, 39 (48%) were referred to their GP for further evaluation, compared with just 23% of the patients audited in 2024.

The results from the current audit are presented in Table 1, which shows that improvements were observed amongst almost all guideline‐based clinical risk factors recorded, when compared with the 2024 audit [3]. The greatest improvement was observed in those patients who were not managed for their osteoporosis risk despite previously documented osteopenia, which fell from 55% to 9% between audits. After introduction of the FRAGILE tool, there was demonstrated improvement in management of patients with a history of poor balance/falls (reduced from 50% to 20%), patients with a history of previous fractures without falls (reduced from 47% to 20%) and patients on specific osteoporosis‐associated medications (reduced from 100% to 57%). Referrals for patients with previous fractures from all mechanisms also experienced a modest improvement, from 30% to 22%. Patients with documented frailty experienced no change (remaining at 14%), whilst patients with specific concomitant diagnosis (e.g., diabetes, rheumatoid arthritis and inflammatory bowel disease) were the only group to experience a reduction in an appropriate referral for osteoporosis screening, with those not managed well rising from 23% to 39% between audits. Overall, these changes resulted in greater recommendations for osteoporosis screening in those patients with more than one of these risk factors, with those failing to be referred dropping from 51% to 24%. The proportion of patients who did not receive an appropriate recommendation for exercise also improved from 26% to 22%.

**TABLE 1:** Management by osteoporosis risk factor, in patients over 50 years of age (274 patients).

Risk factor	Total number	Existing treated osteoporosis	Appropriately managed	Not managed for osteoporosis risk (%)	Not managed in 2024 audit (%)	Improvement from 2024 audit
Previous fracture	23	15	3	5 (22)	7 (30)	29%
Fractures without falls	5	4	0	1 (20)	7 (47)	57%
Risky medications (e.g., steroids)	23	7	3	13 (57)	4 (100)	44%
Age > 70 years	174	64	43	67 (39)	90 (59)	35%
Poor gait balance/falls	79	29	34	16 (20)	28 (50)	59%
Osteopenia	11	9	1	1 (9)	6 (55)	83%
Other risk factors^a^	42	25	7	10 (24)	40 (51)	54%
Documented frailty	7	5	1	1 (14)	1 (14)	0%
Comorbidities (e.g., diabetes)	36	14	8	14 (39)	7 (23)	−67%

*Note:* The values in the table should not be used to calculate totals as many patients had overlapping risk factors.

^a^ ‘Other risk factors’ here include falls/imbalance, immobility, fracture history and drug history.

To further explore factors influencing patient referral for osteoporosis assessment, we performed *χ* ^2^ analyses on three patient‐level variables: age group, sex and disease duration. Patients under 50 years were analysed as a separate group, as minimal trauma fracture in this age group is generally considered indicative of secondary osteoporosis and warrants investigation irrespective of calculated fracture risk. The analysis revealed a statistically significant association between age group and referral rates (*χ* ^2^ (2, *N* = 283) = 11.20, *p* = 0.004). Patients aged over 70 years were more likely to be referred than those aged 50–70 years or under 50 years. In contrast, no significant association was found between sex and referral rates (*χ* ^2^ (1, *N* = 283) = 1.89, *p* = 0.17), indicating that male and female patients were equally likely to be referred when relevant risk factors were identified. The same was true for disease duration and referral rates (*χ* ^2^ (2, *N* = 257) = 3.39, *p* = 0.18), indicating that clinicians did not refer based on disease duration.

## 4 Discussion

Our two‐cycle audit demonstrated that meaningful improvements in screening and referral practices for patients living with Parkinson's and related diseases can occur after implementation of the FRAGILE tool. Referral rates for individuals with relevant risk factors more than doubled between the audits. Thus, even modest interventions, when applied consistently and correctly, can be associated with improved effectiveness of clinical practice.

The improvements observed in this study were not uniform. Referrals still tended to favour more overt risk factors, such as a history of falls/fractures, documented frailty and older age. In contrast, less evident but equally important risk factors, such as concomitant medications and other comorbidities, continued to be overlooked to some extent. This could be attributed to a form of availability bias in which clinicians unconsciously prioritise risks that are more visually salient, commonly encountered or physically associated with the disorder in question, whilst simultaneously under‐recognising subtler or less immediately apparent factors such as medications for comorbid conditions. This phenomenon has been documented in clinical decision‐making literature, particularly when multiple risk factors are present in a time‐constrained setting [6].

Our findings align with the 2022 audit by the UK Parkinson's Excellence Network [7], which reported a similar prevalence of under‐screening and undertreatment of osteoporosis in PD, despite availability of national guidance. A quality improvement project involving 74 UK centres showed that encouraging the use of structured fracture risk tools (e.g., FRAX and QFracture) resulted in a significant improvement in bone health assessment over two audit cycles [8]. However, despite this, studies continued to report substantial variation in how UK PD services assess and manage fracture risk [5, 9], highlighting the persistent gap between clinical knowledge and effective management, even in structured healthcare systems.

The development of the FRAGILE tool was informed by both the RACGP guidelines [4] and literature on common fracture predictors in PD [10, 11] to best improve outcomes in our clinic. However, despite success with the introduction of structured tools in other studies [7, 12], osteoporosis continues to be under‐assessed in PD and related disease patients. This suggests that broader system‐level changes are also necessary, such as developing educational resources aimed at specialist clinicians or working closely with patient advocacy groups to improve education.

Despite the promising nature of the findings, given the study followed the same methodology as our 2024 audit, it is subject to the same limitations and criticisms, including reliance on documentation quality, limited population scope and lack of downstream outcome data, restricting the generalisability of the results [3]. Additionally, although this study measured the outcome of use of the FRAGILE tool, it did not measure the use of the tool itself. This reflected both the nature of a retrospective audit using patient letters as the primary data source, as well as the varied practices of the clinical staff, with both nurses and doctors seeing patients and potentially adopting the tool in different ways. This audit was also potentially subject to other influences, such as general increased awareness of osteoporosis amongst our clinicians between audits. However, we feel that the findings presented here reflect the benefit of the tool in a pragmatic clinic setting. Additionally, the nature and timing of the audit limited our ability to track the ultimate outcome of the GP referral. To better understand the wider applicability and implementation of the tool, an international audit in neurology and geriatrics encompassing multiple centres should be considered.

## 5 Conclusions

This follow‐up audit highlighted the tangible improvements that can be associated with structured clinical recommendations in the assessment and management of osteoporosis risk in PD and related disorders. Although progress has been made, the persistence of gaps in certain risk factor domains, particularly those less intuitively associated with PD and related conditions, underscores the need for ongoing improvement. The proposed FRAGILE tool framework represents a pragmatic step towards standardising osteoporosis screening in this vulnerable population, but broader adoption across multidisciplinary settings will be essential to close the remaining gaps in care.

## Funding

No specific funding was received for this work, but Simon J. G. Lewis is supported by an NHMRC Leadership Fellowship (1195830).

## Ethics Statement

The manuscript does not contain clinical studies or patient data requiring ethical board review. Informed patient consent was not necessary for this work. We confirm that all authors have read the Journal's position on issues involved in ethical publication and affirm that this work is consistent with those guidelines.

## Conflicts of Interest

The authors declare no conflicts of interest.

## Data Availability

The data that support the findings of this study are available from the corresponding author upon reasonable request.
